# Users’ Intrinsic Goals Linked to Alcohol Dependence Risk Level and Engagement With a Health Promotion Website (Hello Sunday Morning): Observational Study

**DOI:** 10.2196/10022

**Published:** 2018-10-22

**Authors:** Emma L Bradshaw, Baljinder K Sahdra, Rafael A Calvo, Alex Mrvaljevich, Richard M Ryan

**Affiliations:** 1 Institute for Positive Psychology and Education North Sydney Australia; 2 University of Sydney Camperdown Australia; 3 Hello Sunday Morning Sydney Australia

**Keywords:** alcohol dependence, aspirations, goals, self-determination theory, website engagement

## Abstract

**Background:**

Hello Sunday Morning (HSM) is a self-guided health promotion website with the aim to improve drinking culture. Members are encouraged to sign up for a 3-month period of alcohol abstention and record and track their progress and goals.

**Objective:**

This study used self-determination theory (SDT) to examine the nature of goals subscribed by HSM users to test the extent to which intrinsic goal pursuit was linked to lower alcohol dependency risk and higher engagement with the HSM website.

**Methods:**

HSM users (N=2216; 59.75%, 1324/2216, females; aged 18-79 years) completed the World Health Organization’s Alcohol Use Disorders Identification Test (WHO-AUDIT, which measures alcohol dependence risk level) at sign-up and at 4 and 6 months after sign-up. In addition, the website had a goals-subscription feature that allowed participants to share their goals. Two independent raters classified the goals according to a coding system we devised based on SDT, which proposes that intrinsic goals (eg, growth, relationships, community, and health) better promote positive outcomes than extrinsic goals (eg, wealth, fame, and image).

**Results:**

Although there was substantial (1016/2216, 45.84%) attrition of HSM users from sign-up to 6 months, the attrition rate could not be attributed to alcohol dependency risk because people in different WHO-AUDIT risk zones were equally likely to be missing at 4 and 6 months after sign-up. The SDT-driven coding of goals yielded the following categories: wealth and image (extrinsic goals); relationships, personal growth, community engagement, and physical health (intrinsic goals); and alcohol use-related goals (which were hard to classify as either extrinsic or intrinsic). Alcohol dependence risk level correlated positively with goals related to money (*r*=.16), personal growth (*r*=.17), relationships (*r*=.10), and alcohol use (*r*=.25). Website engagement correlated negatively with alcohol dependence risk level (*r*=.10) and positively with relationship (*r*=.10) and community goals (*r*=.12).

**Conclusions:**

HSM users with higher alcohol dependence risk tended to engage with the website less, but to the extent that they did, they tended to subscribe to goals related to alcohol use and improving their personal growth, relationships, and finances. In line with SDT, engagement with goals—particularly the intrinsic goals of connecting with close-others and the broader community—related to increased website engagement. Web-based tools intended to promote healthy behaviors in users may be effective in engaging their users if the users’ experience on the website supports the pursuit of intrinsic goals.

## Introduction

The ubiquity of the internet has led to increased interest in its utility for motivating and modifying behavior [1]. Web-based behavior change interventions are usually self-guided programs, designed to promote positive change through the provision of evidence-based materials and interactive online activities [2]. Such programs are often praised for their high reach and low cost, convenience, and the relative anonymity of users [3]. Importantly, these programs are also often effective. Web-based interventions have been useful in decreasing illicit substance use [4], smoking cessation [5], healthy weight management [6], and the promotion of mental health [7].

Web-based interventions may be particularly pertinent to the management of issues related to alcohol consumption, as drinkers tend to prefer self-directed programs such as those provided on the Web [8,9]. Comprehensive reviews indicated that the preference for Web-based programs is high and that they are useful in reducing problematic alcohol consumption [9-11]. Indeed, Web-based alcohol interventions can be as effective as face-to-face interventions [12]. However, the transient nature of Web-surfing renders Web-based programs susceptible to substantial participant dropout or attrition [13-15]. Thus, analysis of the mechanisms that potentially retain and deter users is crucial for maximizing the potential of Web-based behavior change programs.

Web-based platforms can utilize several functionalities to overcome the problem of attrition and motivate behavior change [16]. In particular, “self-monitoring” mechanisms, which facilitate the reporting and tracking of users’ goal progress, are thought to be useful for supporting behavior change goals [16]. The efficacy of these technical goal-tracking mechanisms can also be uniquely informed by psychological theory. Specifically, decades of research suggests that the *types* of goals to which users subscribe may also inform their program engagement.

Self-determination theory (SDT) [17,18] holds that particular goal types relate differentially to positive outcomes. Specifically, some goals are described as intrinsic, referring to goals that reflect inherent growth tendencies and engender intrinsic satisfactions, whereas others are described as extrinsic, as these are focused on outcomes associated with rewards and praise from others [18-21]. Specific intrinsic goals are those that center on personal growth, close relationships, caring for the wider community, and maintaining physical health [19-21]. Conversely, specific extrinsic goals are those for wealth, fame, and esthetic appeal or image [20,21]. Past studies on these distinctions have shown that goal classifications are supported by factor analytic results [19,20,22]. Emphasizing intrinsic relative to extrinsic goals promotes the satisfaction of basic psychological needs (for competence, autonomy, and relatedness), which promotes intrinsic motivation and well-being [19,20,22]. Based on this evidence, a novel contribution to the internet-based intervention research would involve assessing the extent to which an emphasis on intrinsic goals in Web-based environments may encourage engagement and, therefore, strengthen the effects of Web-based interventions.

In this study, we tested the relationship of intrinsic and extrinsic goals with engagement in a sample of participants from the health promotion website Hello Sunday Morning (HSM). With the aim to improve drinking culture, HSM members are encouraged to sign up for a 3-month period of alcohol abstention and, then, connect virtually with like-minded others, blog and post about their experiences, and record and track their goals [23]. HSM employs self-monitoring, as described above, in two main ways.

First, HSM requests that users report their alcohol consumption via weekly reminders and periodic World Health Organization’s Alcohol Use Disorders Identification Test (WHO-AUDIT) [24] completion requests. The WHO-AUDIT is designed to classify respondents into 1 of 4 alcohol consumption risk categories. Scores 0-7 are zone 1 and require simple alcohol education. Simple advice is recommended for those in zone 2 (scores 8-15), which is considered risky or hazardous [23]. Zone 3 members (scores 16-19) are considered engaging in highly risky drinking practices; the suggested intervention is advice plus brief counseling and monitoring. Those in zone 4 (scores 20-40) are thought to be at high risk for alcohol dependency and are best referred to a specialist. Preliminary research suggests that HSM is reaching its target audience, with 95% of participants reporting risky or highly risky drinking practices (meaning they are in zones 2 or 3) and 53% meeting criteria for the likely alcohol dependence (meaning they were in zone 4) [23].

Second, HSM has a goal subscription feature that allows users to specify goals they wish to accomplish. Goals are often alcohol-related, although they do not have to be, and these goals can be selected from a list of popular goals (eg, “Lose weight”), or entered manually if the member has a specific goal in mind. Carah et al [23] examined goals on the HSM platform using a subsample of 2875 participants, reporting that specific goals could be grouped into seven categories: lifestyle, bodywork (comprising fitness and mind and body goals), alcohol, travel, financial, education, relationships, and social. Bodywork and alcohol-related goals were most common, with females more likely than males to sign up for both goal types. Participants with WHO-AUDIT scores >19 (zone 4, likely alcohol dependent) were 2 times more likely to sign up for alcohol-related goals than those with scores between 0 and 7 (zone 1, for whom simple advice and alcohol education is recommended). Rather than using lay categories for goals, we suggest that analyzing intrinsic and extrinsic goals, coded according to the aforementioned principles of SDT, can lead to more specific novel hypotheses regarding website engagement and alcohol consumption, particularly given that evidence suggests that an overemphasis on extrinsic goals relates to increased alcohol consumption [25-27].

Based on the SDT research outlined above, we asked the following Research Question: Do goals (classified according to SDT principles) relate to alcohol dependence risk levels and engagement with the HSM website? Based on the evidence above, goals that center on money, fame, and image may relate to higher alcohol consumption (ie, higher WHO-AUDIT risk level), and members endorsing these goals may be less engaged with the site because of a more controlled, extrinsic orientation. In contrast, growth, relationship, community, and health goals may relate to a lower alcohol risk level, and those endorsing these goals may have better site engagement because of the generally autonomous quality of intrinsic goals [28].

## Methods

### Participants

We examined ongoing WHO-AUDIT completion or noncompletion in a total sample of 2216 HSM users (749 males, 1324 females, and 143 participants did not report gender) with a reported age range of 18-88 (mean 43.59 [SD 15.3]) years at the time of sign-up (845 users did not report the date of birth, as age was not compulsorily reported). They signed up on the HSM website between November 1, 2009, and April 14, 2016. HSM members often complete multiple HSM programs, which consist of completing 3 months alcohol free, but for this study, the researchers had access to data from only those users who completed their first HSM program at sign-up.

As a key focus of this study was the examination of intrinsic and extrinsic goals, we selected only those HSM users who had pertinent goal-related data; this resulted in a sample size of 1117 (age range, 18-88, mean 43.94 [SD 15.09] years). This sample comprised 727 females and 390 males from 30 countries, including Australia (n=399), the United States (n=294), the United Kingdom (n=146), Canada (n=86), New Zealand (n=59), Ireland (n=45), and South Africa (n=12). Of participants, 42 people did not report their country of origin and the remaining 34 were spread across the other 23 countries. All data were collected by HSM and provided in a deidentified format. The website’s terms and conditions stipulate that deidentified data may be used for research purposes. Ethics clearance was obtained from the University of Sydney’s Human Research Ethics Committee (protocol 2016/218).

### Measures

#### Alcohol Dependence Risk Level

We used the widely utilized and validated AUDIT that was developed and recommended by the WHO [24]. The test includes 10 items, 3 designed to measure alcohol consumption and 7 to assess other alcohol-related risks. Example items include “How often do you have 6 or more standard drinks on one occasion?” and “How often during the last year have you felt guilt or remorse after drinking?” each answered on a 5-item scale from 0 (Never) to 4 (Daily or almost daily). Each item is scored from 0 to 4 and then summed into a composite WHO-AUDIT score ranging from 0 to 40, with increasing scores corresponding to escalating risk levels. For those in zone 1 (scores 0-7) and zone 2 (scores 8-15), alcohol education and simple advice is recommended. Zone 3 (scores 16-19) and zone 4 (scores 20-40) suggest alcohol dependence [24,29]. HSM users are invited to complete the WHO-AUDIT when they sign up to the website (T1), 4 months after sign-up (T2), and 6 months after sign-up (T3). The website was programmed to automatically calculate the AUDIT score based on the scoring instructions provided by the WHO, and only the final score was saved in the database.

#### Goals

Participants subscribed to goals (both alcohol-related and nonalcohol-related) ideographically (by typing in their own unique goal) or by selecting from a list of goals prespecified on the HSM platform. The data consisted of 193,465 unique goals; however, many of these goals were similar in their semantic content. To maximize participant counts for each goal, we used title matching in R [30] to search for goals with similar content to that of the most popular goals and merged them. For example, “Get fit and healthy” is a popular goal on HSM, and title matching identified similar goals (with far fewer subscribers) such as “To be more fit and healthy” and “Feel fit and healthy.” Participants subscribing to one of the semantically similar goals, such as the two latter examples included here, were added to the list of participants subscribing to the popular goal. This process yielded 61 popular goals each with at least 100 users listing that goal. Multimedia Appendix 1 provides the list of these goals.

#### Engagement

Participant engagement was operationalized through the calculation of a cumulative count of every participation occasion [31]. An engagement count was earned every time a participant engaged with the site; this includes passive actions, such as simply logging into their account, and active actions, such as commenting on another user’s post, posting a comment, liking and be-friending other members.

## Results

### Attrition Over Time

We examined attrition on HSM over time as a function of WHO-AUDIT scores. Multimedia Appendix 2 presents individual trajectories of participants’ alcohol risk levels from sign-up up to 6 months after sign-up. Evidently, there was individual variation, as well as attrition, over time. Indeed, of the participants who were available at T1 (sign-up), 65% (1434/2216) were present at T2 (4 months after sign-up) and 54% (1200/2216) at T3 (6 months after sign-up). Table 1 reports the mean and median scores for each alcohol dependence risk zone at sign-up and at 4 and 6 months after sign-up. While WHO-AUDIT scores at T1 were normally distributed, the distributions of the scores at all subsequent waves were skewed such that those in zones 1 and 2 (the lower-risk categories) appeared most frequently (see Multimedia Appendix 3 for WHO-AUDIT frequency distribution plots at each time-point).

We did not conduct any longitudinal statistical models of these data (eg, to test the change in WHO-AUDIT scores over time) because the results would yield biased estimates because of the high attrition observed in the data, but we did examine the extent to which attrition was systematically linked to WHO-AUDIT scores. For instance, we assessed whether people in higher- than lower-risk zones would be more likely to be absent than present at later waves of data. Table 2 depicts the percentage of available and absent participants in each WHO-AUDIT zone at 4 and 6 months after sign-up.

**Table 1:** World Health Organization’s Alcohol Use Disorders Identification Test (WHO-AUDIT) means, SDs, and medians for each alcohol dependence risk zone at sign-up (T1) and 4 (T2) and 6 months (T3) after sign-up.

Zones^a^	T1		T2		T3	
	Mean (SD)	Median	Mean (SD)	Median	Mean (SD)	Median
Zone 1	4.10 (2.10)	4	1.87 (2.44)	1	1.92 (3.02)	1
Zone 2	11.92 (2.25)	12	5.19 (5.59)	3	4.99 (4.58)	4
Zone 3	17.52 (1.08)	17.5	6.93 (6.78)	5	7.48 (7.08)	6
Zone 4	25.39 (4.23)	25	10.12 (10.02)	7	10.90 (10.02)	8

^a^Zone 1 (low risk), AUDIT scores 0-7; Zone 2 (moderate risk of harm), AUDIT scores 8-15; Zone 3 (high risk), AUDIT scores 16-19; and Zone 4 (almost certainly dependent), AUDIT scores 20-40.

**Table 2:** The percentage of participants present and missing in each alcohol risk zone 1 month (Time 2) and 3 months (Time 3) after sign-up.

Time-point		Zone^a^ 1	Zone^a^ 2	Zone^a^ 3	Zone^a^ 4
**Time 2**					
	Present	70.97	65.38	66.75	62.61
	Missing	29.03	34.62	33.25	37.39
**Time 3**		54.30	55.47	52.96	54.11
	Present	54.30	55.47	52.96	54.11
	Missing	45.70	44.53	47.04	45.89

^a^Zone 1 (low risk), AUDIT scores 0-7; Zone 2 (moderate risk of harm), AUDIT scores 8-15; Zone 3 (high risk), AUDIT scores 16-19; and Zone 4 (almost certainly dependent), AUDIT scores 20-40.

A nonparametric test of the differences in missing and nonmissing participants in each risk zone at different waves was not significant (*χ*^2^_3_=0.6, *P*=.90), indicating that attrition was not linked to the alcohol risk level because members of the 4 alcohol risk zones were equally likely to be present (or missing) in any given wave.

### Self-Determination Theory-Informed Analysis of Goals

The 61 most popular goals were categorized according to SDT [18] principles as implemented in the Aspiration Index [20], which distinguishes extrinsic goals (wealth, fame, and image or attractiveness) from intrinsic goals (personal growth, close relationships, community service, and physical health). Accordingly, we classified the goals into one of the 7 aspirations categories. Five goals were coded as money-related (285 counts), examples included “Get out of debt” and “Save money” (notably, the finance-related goals did not center on the acquisition of abundant wealth as specified in the Aspiration Index, as such these goals have been relabeled “money” goals as opposed to wealth). As no fame goals were identified, this category was omitted from the analysis. Two image goals (614 counts) emerged, including “Lose weight” and “Get my body mass index under 25,” which were both double-coded with physical health. In addition, there were 15 personal growth goals (893 counts) such as “Find a new hobby,” as well as 11 relationship goals (550 counts), including “Treat my partner better,” 3 community goals (19 counts), including “Volunteer in a cause greater than myself, even for one day,” and 13 physical health goals (1869 counts), for example, “Get fit and healthy” and “Stop smoking.” A separate category of alcohol-related goals was created to encapsulate the popular goals for which an intrinsic or extrinsic orientation could not be inferred. Furthermore, 29 goals were coded as alcohol-related (1907 counts), for example, “Dance sober” and “Have a sober new year’s.”

Two raters independently coded the 61 goals into the seven goal categories. An interrater reliability analysis using Cohen kappa was performed to determine the interrater consistency. Cohen kappa was .78 for relationships; .86 for personal growth; .97 for alcohol; and 1 for money, image, community, and physical health, suggesting substantial to perfect agreement [32]. These goal-level analyses provided initial evidence that a theory-driven approach to categorizing goals was informative—participants’ goals can be meaningfully categorized in this manner with independent raters showing a sufficiently high degree of agreement. To conduct participant-level analyses of the goals, the goals codes were summed to create a per-participant count for each of the seven goal categories. For instance, a participant could potentially get a count of 0 for money but 5 for physical health and so on. The seven goal variables, therefore, represented count data.

### Intercorrelations

Table 3 provides Spearman correlations, means, and SDs of the goals count variables with the count data of engagement and the initial WHO-AUDIT scores at sign-up. The Spearman method uses ranks and is more conservative than Pearson’s *r*, which is appropriate, given our use of count data and the large sample size.

**Table 3:** Spearman correlations, means, and SDs of study variables.

Study variables	Mean (SD)	1^a^	2^b^	3^c^	4^d^	5^e^	6^f^	7^g^	8^h^
WHO-AUDIT^i^	18.95 (7.87)								
Money	0.26 (0.47)	0.16^j^							
Image	0.55 (0.54)	0.01^k^	0.05^k^						
Growth	0.80 (0.75)	0.17^j^	0.10^j^	0.03^k^					
Relationships	0.49 (0.71)	0.10^j^	0.18^j^	0.00^k^	0.17^j^				
Community	0.02 (0.14)	−0.02^k^	−0.02^k^	−0.08^k^	0.02^k^	0.06^k^			
Health	1.67 (1.09)	0.05^k^	0.18^j^	0.66^j^	0.12^j^	0.10^j^	−0.10^j^		
Alcohol	1.71 (1.37)	0.25^j^	0.22^j^	0.10^j^	0.58^j^	0.57^j^	0.04^k^	0.20^j^	
Engagement	36.37 (41.96)	−0.10^j^	−0.03^k^	−0.01^k^	0.00^k^	0.10^j^	0.12^j^	−0.03^k^	0.04^k^

^a^1: World Health Organization Alcohol Use Disorders Identification Test at the sign-up time-point (T1).

^b^2: money-related goal counts.

^c^3: image-related goal counts.

^d^4: personal growth-related goal counts.

^e^5: relationship-related goal counts.

^f^6: community service-related goal counts.

^g^7: physical health-related goal counts.

^h^8: alcohol-related goal counts.

^i^WHO-AUDIT: World Health Organization’s Alcohol Use Disorders Identification Test.

^j^*P*<.05 with a 95% CI.

^k^Nonsignificant.

Alcohol-related goals correlated positively with all other goals, except those related to the community (for which goal counts were low), indicating that reducing consumption is a common thread throughout HSM users’ goal activity. While the intrinsic goals for money and image are positively correlated in the aspirations literature [20], these goals were not correlated in these data. Indeed, image goals related only to alcohol goals and health goals, suggesting that image goals may have a more intrinsic quality on the HSM platform, thus explaining their link to physical health and absence of such a link to money goals. Within the intrinsic domain, all goals were positively correlated, again except for community goals, which is largely congruent with the existing literature [19,20,22]. Money, growth, relationships, and—intuitively—alcohol goals related positively with alcohol dependence risk level, meaning that as alcohol dependence risk level increased so did subscription to these goals; this result aligns with evidence suggesting that extrinsic goals relate to increased alcohol consumption [25], though links to growth and relationships are unexpected. Alcohol dependence risk level did not relate to image, community, or health goals. Moreover, engagement related positively to relationship- and community-related goals, and, interestingly, engagement had a statistically significant, weak negative correlation with alcohol dependence risk level. This result indicates that HSM users with higher alcohol dependence risk tended to engage with the website less.

## Discussion

To inform research on potential mechanisms of participant engagement in Web-based interventions, in this study, we used an SDT-driven approach to analyze participants’ goals on the HSM platform. Based on the theory [18,20], we predicted that intrinsic goals (goals related to personal growth, relationships, community, and health) would relate to increased website engagement and that the endorsement of extrinsic goals (goals related to money and image) would relate negatively to engagement and predict alcohol dependence risk level (WHO-AUDIT scores). The engagement hypotheses were partly supported by the positive correlation between relationship and community goals and engagement, although engagement was not related to either extrinsic goal. WHO-AUDIT scores related positively to extrinsic goals related to money, though not to image goals, also partly supporting our alcohol risk level hypothesis. Counter to our expectations, WHO-AUDIT scores also positively correlated with personal growth and relationship goals, both of which are intrinsic.

Our initial analysis also identified some participant attrition (noncompletion of WHO-AUDIT assessments) from the sign-up time-point through the two follow-ups, which is consistent with past evidence suggesting that hazardous alcohol consumption predicts dropout in Web-based interventions [12]. Of the initial 2216 users, 35.29% (782/2216) were missing at T2 and 45.85% (1016/2216) were missing at T3; these participation rates are respectable given that dropouts for Web-based social network health behavior interventions more generally tend to be in excess of 80% [33]. Rates of attrition were not different across alcohol dependence risk zones, which indicates that dropout (or retention) was equally likely in each of the 4 risk zone groups; this result was unexpected, given that heavy drinkers are often the least amenable to intervention and are prone to program noncompletion [8,12]. While higher risk participants were no less likely to report alcohol dependence risk level over time (as indicated by the WHO-AUDIT reporting results discussed above), they were engaging with the site less, as evident from the negative correlation between engagement and alcohol dependence. Notably, the correlation, while statistically significant, was weak (*r*=−.10), accounting for 1% of the shared variance between engagement and the WHO-AUDIT.

Consistent with SDT, relationship- and community-related goals related positively to engagement; this may suggest that engagement with more intrinsic, socially oriented goals promotes platform engagement. Arguably, this is because intrinsic aspirations are thought to demonstrate more of an other-orientation than a self-orientation [34], and interactive Web-based environments such as HSM present opportunities to connect and interact with other members.

The correlations with the seven goal count variables indicate that high endorsement of alcohol-, money-, growth-, and relationships-related goals may predict a higher risk of alcohol dependency (eg, higher WHO-AUDIT scores). The relationship of alcohol-related goals to alcohol dependence risk level is intuitive; the more at risk one is, the more their goals might center on reducing that risk. With regards to the correlation between the WHO-AUDIT and money-related goals, an extrinsic aspirational focus has been found to relate to increased alcohol consumption [25]; in addition, and more pragmatically, the more one drinks, the more one is likely to spend on alcohol, so this relationship is evidentially and practically founded. However, the positive correlation between the WHO-AUDIT and growth and relationship goals is unexpected. Growth goals included, for example, “Find a new hobby,” and an example relationship goal is to “Treat my partner better”; the theoretical link between goals of this nature and alcohol dependence risk level is unclear and may simply be a function of the fact that this sample is, on average, at high alcohol dependence risk level relative to the general population.

The strengths of this study lay in its large sample size and the combination of qualitative and quantitative methodologies. However, the analysis was limited by several factors. We were not able to model the data longitudinally because of the attrition rate. Furthermore, we were not able to test the internal consistency of the WHO-AUDIT in this sample because of the inability to access the individual item responses. However, the fact that WHO-AUDIT scores correlate meaningfully with other relevant variables is some evidence of its construct validity. In addition, this study is, of course, not an efficacy study. The systematic examination of alcohol risk level and participant engagement using a randomized controlled trial is a logical next step for future research.

Despite these limitations, this study illustrates that an SDT-driven approach can lead to new insights and a better understanding of users’ experiences in Web-based behavior change environments such as HSM. The theory-congruent and novel results presented here set the scene for future replication and expansion using longitudinal datasets. Hence, subscription to intrinsic, more socially oriented goals may help users remain engaged with such health promotion platforms.

## Appendix

Multimedia Appendix 1 List of the top 61 goals.

Multimedia Appendix 2 Individual trajectories of alcohol risk over time measured by the World Health Organization’sAlcohol Use Disorders Identification Test (WHO-AUDIT).

Multimedia Appendix 3 Distribution of alcohol risk level as measured by the World Health Organization’s Alcohol Use Disorders Identification Test (WHO-AUDIT).

## Abbreviations

HSM: Hello Sunday Morning
SDT: self-determination theory
WHO-AUDIT: World Health Organization’s Alcohol Use Disorders Identification Test

## References

[1] Elaheebocus SMRA, Weal M, Morrison L, Yardley L (2018). Peer-Based Social Media Features in Behavior Change Interventions: Systematic Review. J Med Internet Res.

[2] Barak A, Klein B, Proudfoot JG (2009). Defining internet-supported therapeutic interventions. Annals of Behavioral Medicine.

[3] Santarossa S, Kane D, Senn CY, Woodruff SJ (2018). Exploring the role of in-person components for online health behavior change interventions: Can a digital person-to-person component suffice?. J Med Internet Res.

[4] Boumparis N, Karyotaki E, Schaub MP, Cuijpers P, Riper H (2017). Internet interventions for adult illicit substance users: A meta-analysis. Addiction.

[5] Graham AL, Carpenter KM, Cha S, Cole S, Jacobs MA, Raskob M, Cole-Lewis H (2016). Systematic review and meta-analysis of Internet interventions for smoking cessation among adults. Substance Abuse and Rehabilitation.

[6] Sorgente A, Pietrabissa G, Manzoni GM, Re F, Simpson S, Perona S, Rossi A, Cattivelli R, Innamorati M, Jackson JB, Castelnuovo G (2017). Web-based interventions for weight loss or weight loss maintenance in overweight and obese people: A systematic review of systematic reviews. J Med Internet Res.

[7] Nguyen-Feng V, Greer CFP, Frazier P (2017). Using online interventions to deliver college student mental health resources: Evidence from randomized clinical trials. Psychological Services.

[8] Black DR, Coster DC (1996). Interest in a stepped approach model (SAM): identification of recruitment strategies for university alcohol programs. Health Education Quarterly.

[9] Ganz T, Braun M, Laging M, Schermelleh-Engel K, Michalak J, Heidenreich T (2018). Effects of a stand-alone web-based electronic screening and brief intervention targeting alcohol use in university students of legal drinking age: A randomized controlled trial. Addictive Behaviors.

[10] Sundström C, Blankers MKZ, Khadjesari Z (2017). Computer-based interventions for problematic alcohol use: A review of systematic reviews. International Journal of Behavioral Medicine.

[11] Riper H, Blankers M, Hadiwijaya H, Cunningham J, Clarke S, Wiers R, Ebert D, Cuijpers P (2014). Effectiveness of guided and unguided low-intensity internet interventions for adult alcohol misuse: a meta-analysis. PLoS One.

[12] Radtke T, Ostergaard M, Cooke R, Scholz U (2017). Web-Based Alcohol Intervention: Study of Systematic Attrition of Heavy Drinkers. J Med Internet Res.

[13] Ahern DK (2007). Challenges and opportunities of eHealth research. American Journal of Preventive Medicine.

[14] Habibović M, Cuijpers P, Alings M, van der Voort Pepijn, Theuns D, Bouwels L, Herrman J-P, Valk S, Pedersen S (2014). Attrition and adherence in a WEB-Based Distress Management Program for Implantable Cardioverter defibrillator Patients (WEBCARE): randomized controlled trial. J Med Internet Res.

[15] Sawyer M, Reece CE, Bowering K, Jeffs D, Sawyer ACP, Peters JD, Mpundu-Kaambwa C, Clark JJ, McDonald D, Mittinty MN, Lynch JW (2016). Usage, adherence and attrition: How new mothers engage with a nurse-moderated web-based intervention to support maternal and infant health. A 9-month observational study. BMJ Open.

[16] Lehto TOH, Oinas-Kukkonen H (2011). Persuasive features in web-based alcohol and smoking interventions: A systematic review of the literature. J Med Internet Res.

[17] Deci EL, Ryan RM (1985). Intrinsic motivation and self-determination in human behavior.

[18] Ryan RM, Deci EL (2017). Self-determination theory: Basic psychological needs in motivation, development and wellness.

[19] Kasser T, Ryan RM (2016). Further examining the American dream: Differential correlates of intrinsic and extrinsic goals. Personality and Social Psychology Bulletin.

[20] Kasser T, Ryan RM, Schmuck P, Sheldon K (2001). Be careful what you wish for: Optimal functioning and the relative attainment of intrinsic and extrinsic goals. Life goals and well-being: Towards a positive psychology of human striving.

[21] Unanue W, Vignoles VL, Dittmar H, Vansteenkiste M (2016). Life goals predict environmental behavior: Cross-cultural and longitudinal evidence. Journal of Environmental Psychology.

[22] Kasser T, Ryan RM (1993). A dark side of the American dream: Correlates of financial success as a central life aspiration. Journal of Personality and Social Psychology.

[23] Carah N, Meurk C, Hall W (2015). Profiling Hello Sunday Morning: who are the participants?. International Journal of Drug Policy.

[24] Higgins-Biddle JC, Babor TF (2001). Brief intervention for hazardous and harmful drinking: A manual for use in primary care.

[25] Shamloo ZS, Cox WM (2010). The relationship between motivational structure, sense of control, intrinsic motivation and university students' alcohol consumption. Addictive Behaviors.

[26] Sheldon KM, Krieger LS (2014). Service job lawyers are happier than money job lawyers, despite their lower income. The Journal of Positive Psychology.

[27] Williams G, Hedberg VA, Cox EM, Deci EL (2000). Extrinsic life goals and health-risk behaviors in adolescents. Journal of Applied Social Psychology.

[28] Kasser T, Deci EL, Ryan RM (2002). Sketches for a self-determination theory of values. Handbook of self-determination research.

[29] Rubinsky AD, Kivlahan DR, Volk RJ, Maynard C, Bradley KA (2010). Estimating risk of alcohol dependence using alcohol screening scores. Drug and Alcohol Dependence.

[30] (2015). R Core Team.

[31] Kelders SM, Kok RN, Ossebaard HC, Van Gemert-Pijnen JEWC (2012). Persuasive system design does matter: A systematic review of adherence to web-based interventions. J Med Internet Res.

[32] Landis JR, Koch GG (1977). The Measurement of Observer Agreement for Categorical Data. Biometrics.

[33] Maher CA, Lewis LK, Ferrar K, Marshall S, De Bourdeaudhuij I, Vandelanotte C (2014). Are health behavior change interventions that use online social networks effective? A systematic review. J Med Internet Res.

[34] Weinstein N, Przybylski AK, Ryan RM (2009). Can nature make us more caring? Effects of immersion in nature on intrinsic aspirations and generosity. Personality and Social Psychology Bulletin.

