# Supplementary material for: Users’ Intrinsic Goals Linked to Alcohol Dependence Risk Level and Engagement With a Health Promotion Website (Hello Sunday Morning): Observational Study
Source: JMIR Ment Health. 2018 Oct 22;5(4):e10022. doi: 10.2196/10022 (PMC6231871; doi:10.2196/10022)
Supplement: Multimedia Appendix 1 [file mental_v5i4e10022_app1.pdf]

S1

List of the top 61 goals

Find alternate way to wind down at home without alcohol
Regain focus, concentration and energy
Complete a 3-month HSM!
Get fit and healthy
Exercise 5 days a week
Stop drinking alone
Make new sober friends
Get out of debt
DON'T DRINK TODAY
Go 1 week alcohol free
30 days alcohol free
Experience my feelings without alcohol
Be a role model for my children
Quit drinking forever
Be happier and more positive
Learn to drink in moderation
Treat my partner better
Being able to socialize whilst being sober with people who are drinking
Drink more water
Save the money that would have been spent on alcohol.
Stop Smoking
No more hangovers!
Be as fit and healthy as I can be
Lose weight
Learn to socialise without drinking
Save money
Attend yoga twice a week
Have a sober birthday
Get 8 hours sleep a night
Start enjoying my life
Learn to laugh more, smile more and be less sensitive.
Write down things that I'm grateful for every week
Have fun at parties without alcohol
Read High Sobriety
Be a better and more reliable friend

Post every Sunday on Hello Sunday Morning
Be more active on the weekends
Run a half marathon
Have a sober new years
Holiday without alcohol
Moderate drinking Friday and Saturday's
Find a new hobby
Read 5 books
To spend more time with my family instead of drinking
Develop a budget and stick to it
Get through a work function without booze
Cook a new recipe every week
Save money for a holiday
Get my BMI under 25
Dance sober
Keep track of the number of drinks I have everyday
Learn a new language
Have less than 10 drinks a week
Tell people around me that I'm doing a HSM
To spread awareness on the dangers of drink driving
Run 5km in under 40 minutes
Find a new job
Graduate from university
Go on a first date sober
Blog or support a fellow HSM-er every day
Volunteer in a cause greater than myself, even for one day
