# Supplementary material for: Users’ Intrinsic Goals Linked to Alcohol Dependence Risk Level and Engagement With a Health Promotion Website (Hello Sunday Morning): Observational Study
Source: JMIR Ment Health. 2018 Oct 22;5(4):e10022. doi: 10.2196/10022 (PMC6231871; doi:10.2196/10022)

S2

The individual participant trajectories of alcohol risk level as measured by the World Health Organisation's Alcohol Use Disorders Identification Test (WHO-AUDIT) from the sign-up time point (Time 1), four months after sign-up (Time 2), and six months after sign up (Time 3).

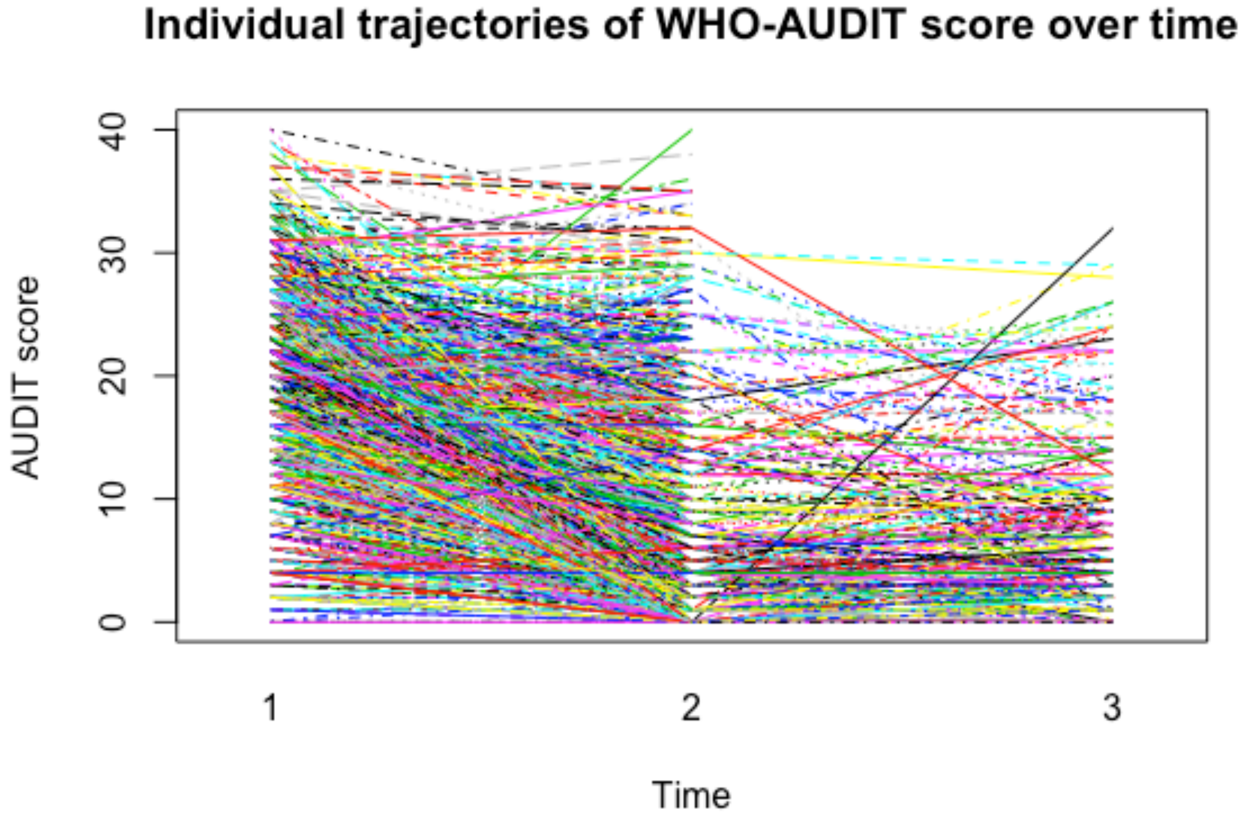

Supplement: Multimedia Appendix 2 [file mental_v5i4e10022_app2.pdf]
